# Alleviatory effects of silicon on the foliar micromorphology and anatomy of rice (*Oryza sativa* L.) seedlings under simulated acid rain

**DOI:** 10.1371/journal.pone.0187021

**Published:** 2017-10-24

**Authors:** Shuming Ju, Liping Wang, Cuiying Zhang, Tingchao Yin, Siliang Shao

**Affiliations:** 1 School of Environment and Spatial Informatics, China University of Mining & Technology, Xuzhou, Jiangsu, China; 2 School of Environment, Xuzhou Institute of Technology, Xuzhou, Jiangsu, China; 3 Jiangsu Laboratory of Pollution Control and Resource Reuse, Xuzhou, Jiangsu, China; Henan Agricultural University, CHINA

## Abstract

Silicon (Si) is a macroelement in plants. The biological effects and mitigation mechanisms of silicon under environmental stress have become hot topics. The main objectives of this study were to elucidate the roles of Si in alleviating the effects on the phenotype, micromorphology and anatomy of the leaves of rice seedlings under acid rain stress. The results indicated that the combined or single effects of Si and simulated acid rain (SAR) stress on rice roots depended on the concentration of Si and the intensity of the SAR stress. The combined or single effects of the moderate concentration of Si (2.0 mM) and light SAR (pH 4.0) enhanced the growth of the rice leaves and the development of the mesophyll cells, and the combined effects were stronger than those of the single treatments. The high concentration of Si (4.0 mM) and severe SAR (pH 3.0 or 2.0) exerted deleterious effects. The incorporation of Si (2.0 or 4.0 mM) into SAR at pH values of 3.0 or 2.0 promoted rice leaf growth, decreased necrosis spots, maintained the structure and function of the mesophyll cells, increased the epicuticular wax content and wart-like protuberance (WP) density, and improved the stomatal characteristics of the leaves of rice seedlings more than the SAR only treatments. The alleviatory effects observed with a moderate concentration of Si (2.0 mM) were better than the effects obtained with the high concentration of Si (4.0 mM). The alleviatory effects were due to the enhancement of the mechanical barriers in the leaf epidermis.

## Introduction

Silicon (Si) is a beneficial macroelement in plants [1–4]. Many studies have shown that the application of silicon can enhance the resistance of plants exposed to abiotic and biotic stresses, such as acid rain [5], UV-B [6], drought [7,8], metal toxicity [9], salt [8], diseases [10,11] and others [12]. Si is taken up by the roots as silicic acid, and the silicic acid is transported to the shoot through the xylem and then undergoes polymerization to hydrated biogenic silica (SiO_2_·nH_2_O) [13]. Si accumulates primarily in epidermal cells, stomata, trichomes in leaves and transfusion tissue, and increases in Si can thicken and strengthen the cell wall [14–16]. The abaxial epidermis of rice leaves has an average of 1.5 times more Si than the adaxial epidermis [16]. Improving the organization structure is recognized as a mechanism through which Si application relieves environmental stresses [17–19]. The addition of exogenous Si has been found to alter the stomata structure in begonia and pansy plants, which decreases the deleterious effects caused by salinity stress [20]. The application of Si to plants under drought stress could enhance epicuticular wax for mation [21].

Morphological structure changes are an adaptation and resistance mechanism of plants to environmental stresses. The epidermis of plants is the first barrier to environmental stress and acts as a mode of self-preservation in plants. The leaf is an assimilative organ and is the largest and most significant part of the plant that is exposed to the environment. Therefore, the improvement of leaf epidermis characteristics represents an important defence strategy. The leaf is the most sensitive organ to acid rain and has been the target of previous acid rain research [22–27]. Acid deposition can induce severe damage to epidermal tissues, causing alterations in the cuticular wax layer and the leaf surface morphology and may even lead to cellular death [22,28–30]. According to the above research and analyses, we assume that supplying Si to plants under acid rain stress may induce changes in the characteristics of the leaf epidermis that could alleviate acid rain damage.

Rice is the second leading food crop worldwide [31,32] and is a typical Si-hyperaccumulating plant species. Moreover, rice is commonly grown in acid rain regions, including China [33]. Previous studies have shown that Si applications to rice improved the growth; decreased the malondialdehyde (MDA) and H_2_O_2_ content; increased CAT, POD, SOD, and APX activities; maintained the balance of K, Ca, Mg, Fe, Zn, Cu mineral elements; and promoted photosynthesis. The influence of the supply of Si on rice seedlings was found to depend on the Si concentration, and a moderate concentration of Si (2.0 mM) was more beneficial than a low or high concentration of Si (1.0and 4.0 mM) [5,34]. The present study investigated the effects of Si on the leaf phenotype, mesophyll cell structure, Si content, stoma characteristics, epicuticular wax and wart-like protuberance (WP) characteristics of rice seedlings under simulated acid rain (SAR) stress. The investigation aimed to understand how the supply of Si leads to changes in the organizational structure of the leaf epidermis of rice seedlings exposed to acid rain. Moreover, this study discusses the mechanisms and structure of Si-mediated alleviation of acid rain stress and provides evidence demonstrating the roles of Si in enhancing the tolerance of rice seedlings to abiotic stress.

## Materials and methods

### Plant materials and experimental designs

Rice seedlings were cultured as described in our previous study [5,34]. Rice seeds (Zhendao 95, Xuzhou Seed Co., Ltd., Xuzhou Jiangsu, China) were germinated in an incubator at 25 ± 1°C. 6-day-old seedlings were transferred to 1/4-strength International Rice Research Institute (IRRI) solution in a greenhouse at 25 ± 5°C. Then, 15-day-old seedlings and 24-day-old seedlings were cultured in 1/2-strength and full-strength IRRI solutions, respectively [5,34,35]. The nutrient solution was renewed every 3 days to stabilize the pH value, and water was added every day to maintain the solution volume. The rice nutrients (IRRI solution), SAR (pH 2.0, 3.0 and 4.0) and Si solutions (2 and 4mM) were prepared according to previously reported methods [5,34]. The pH of the nutrient solution was adjusted to 5.5 with 1 M NaOH or HCl using a PHS-29A pH meter (Shanghai Anting Scientific Instrument Factory, Shanghai, China). To obtain the 1/4- and 1/2-strength nutrient solutions, the macroelement concentrations were decreased to values equal to 1/4 and 1/2 of the initial values, respectively; however, the Ca concentration was not changed. 25-day-old seedlings were selected to be sprayed with acid rain. The SAR solution was adjusted to 6.5(control), 4.0, 3.0, and 2.0 using 1 mM H_2_SO_4_ and 1 mM HNO_3_ at a ratio of 2.7:1 by the chemical equivalents [36,37]. The pH value [38,39] and the ionic composition of acid rain in China was derived from precipitation data from eastern China [40,41]. The SAR solutions were prepared as described in our previous study [5,34]. The Si treatments were initiated when the seedlings were six days old; the 0, 2 and 4 mM Si solutionswere prepared by dissolving appropriate quantities of Na_2_SiO_3_·9H_2_O in the IRRI nutrient solution.

A full factorial experimental design was used with 12 total treatment combinations. (1) The control treatment: rice seedlings were cultured in IRRI nutrient solution without Si (0 mM) and then sprayed with SAR (pH 6.5). (2) Single Si treatment: rice seedlings were cultured in IRRI nutrient solution with Si (2 or 4 mM, pH 5.5) and then sprayed with SAR (pH 6.5). (3) Single SAR treatment: rice seedlings were cultured in IRRI nutrient solution without Si (pH 5.5) and then sprayed with SAR (pH 4.0, 3.0 or 2.0). (4) Combined Si and SAR treatment: rice seedlings were cultured in IRRI nutrient solution with Si (2 or 4 mM, pH 5.5) and then sprayed with SAR (pH 4.0, 3.0 or 2.0). The spraying of the SAR solution on the leaf continued until drops began to fall off the foliage. Three replicates were performed for each treatment. All plants were grown with a natural photoperiod, and the relative humidity was controlled at 50% to 70%. The rice seedlings were treated with SAR for 7 days and were collected to determine the test indices.

### Transmission electron microscopy (TEM) observation

The ultrastructure of the mesophyll cells in the rice seedlings was observed under TEM according to modified previous methods [42]. The middle sections of the 2^nd^ set of fresh leaves were used for the TEM observations. The leaves were cut into 1.0×2.0mm pieces. After fixation with 4% glutaraldehyde (2 M, pH 7.2) for 24 h, the leaf cells were fixed with 2% osmic acid at 4°C for 2 h and then dehydrated using an ethanol gradient (30%, 50%, 70%, 90%, 100%). The samples were then embedded in 100% Epon-812 and polymerized at 80°C for 24 h. The samples were cut into ultrathin sections using anLKB-V ultramicrotome (Bromma, Stockholm, Sweden) for ultrastructural observations. The ultrathin sections were then passed through 250-mesh grids and post-stained with uranyl acetate and lead citrate. Finally, TEM images were obtained using a transmission electron microscope (JEM-2100, JEOL, Japan).

### Scanning electron microscopy (SEM) observation and energy dispersive spectroscopy (EDS) analysis

Methods modified from Li et al. [43] were primarily used in this study. The middle sections of the 2^nd^set of fresh leaves were prepared for SEM and EDS analyses. The leaves were cut into 5.0×5.0mm pieces. After freeze drying, the samples were adhered to the sample table then coated with gold using anion sputter apparatus (MODEL E-1010HITACHI ION SPUTTER JEOL, Japan) at a current of 15 mA and a time of 50s. The morphological structure of the epidermis of the leaf blade was examined using SEM (FEI QUANTA 200, ENVIRONMENTAL SCANNING ELECTRON MICROSCOPE, Netherlands). The relative silicon content was determined using EDS (INCA ADDE-250, OXFORD, UK) combined with SEM at an accelerating voltage of 20 kV and a distance of 10.0 mm.

### Statistical analysis

The means ± standard deviations were calculated using SPSS 19.0. One-way analysis of variance (ANOVA) with Tukey’s honestly significant difference (HSD) was used to analyse significant differences between treatments, and two-way ANOVA was performed to test the interaction between Si and SAR using SPSS19.0. Correlation analysis was performed to investigate the relationship of Si content and wart-like protuberance (WP) number in leaves epidermis using Origin 8.0.

## Results

### Combined effects of Si and SAR on the leaves of rice seedlings

**Fig 1:**
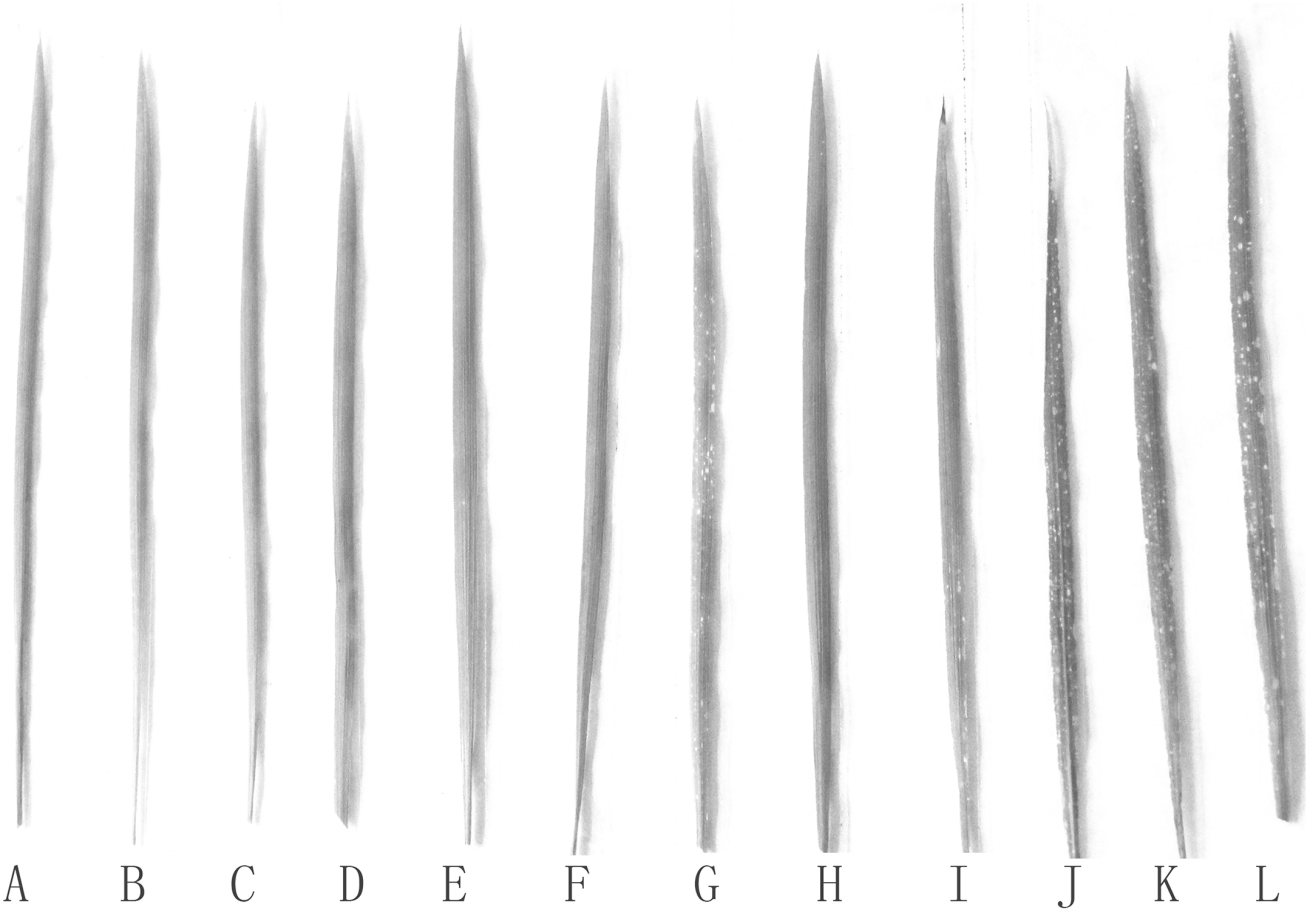
Symptom of rice leaves under different treatments: (A-C) rain at pH 6.5; (D-F) SAR at pH 4.0; (G-I) SAR at pH 3.0; (J-L) SAR at pH 2.0; (A, D, G, J) no Si; (B, E, H, K) 2 mM Si; and (C, F, I, L) 4 mM Si. Photographs were taken 1 week after the final SAR treatment.

The phenotypic images of the leaves of rice seedlings treated with Si and SAR are displayed in Fig 1A–1L. The leaves of the seedlings subjected to the 2 mM Si treatments were notably different (Fig 1B). However, the leaves of the rice seedling streated with 4 mM Si were smaller than those of the control (Fig 1C). The leaves of the rice seedlings subjected to the SAR treatments were markedly different from those of the control. The leaves treated with the SAR at pH 4.0 were larger than the control leaves (Fig 1D); the leaves of the seedlings treated with SAR at pH 3.0 also exhibited changes and had more small necrosis spots compared with the control (Fig 1G), and the leaves treated with SAR at pH 2.0 were more severely yellowed, and necrosis was observed on the whole leaves (Fig 1J).

The combined 2 mM Si and SAR at pH 4.0 treatments revealed better symptoms than those of the control, the treatment with the single 2 mM Si and the treatment with the single SAR pH at 4.0 (Fig 1E). The leaves treated with 4 mM Si and SAR at pH 4.0were in better condition than those of the single 4.0 mM Si treatment (Fig 1F). The leaves treated with 2 or 4 mM Si and SAR at pH 3.0 or 2.0 all exhibited necrosis spots. The incorporation of exogenous 2 or 4 mM Si into the SAR at pH 3.0 or 2.0 significantly decreased the number of necrosis spots and improved the resistance compared with the treatment with only SAR at pH 3.0 or 2.0 (Fig 1H, 1I, 1K and 1L), respectively. Moreover, the incorporation of exogenous 2 mM Si revealed better results than that of the exogenous 4 mM Si treatment.

### Combined effects of Si and SAR on the mesophyll cells of rice seedlings

**Fig 2:**
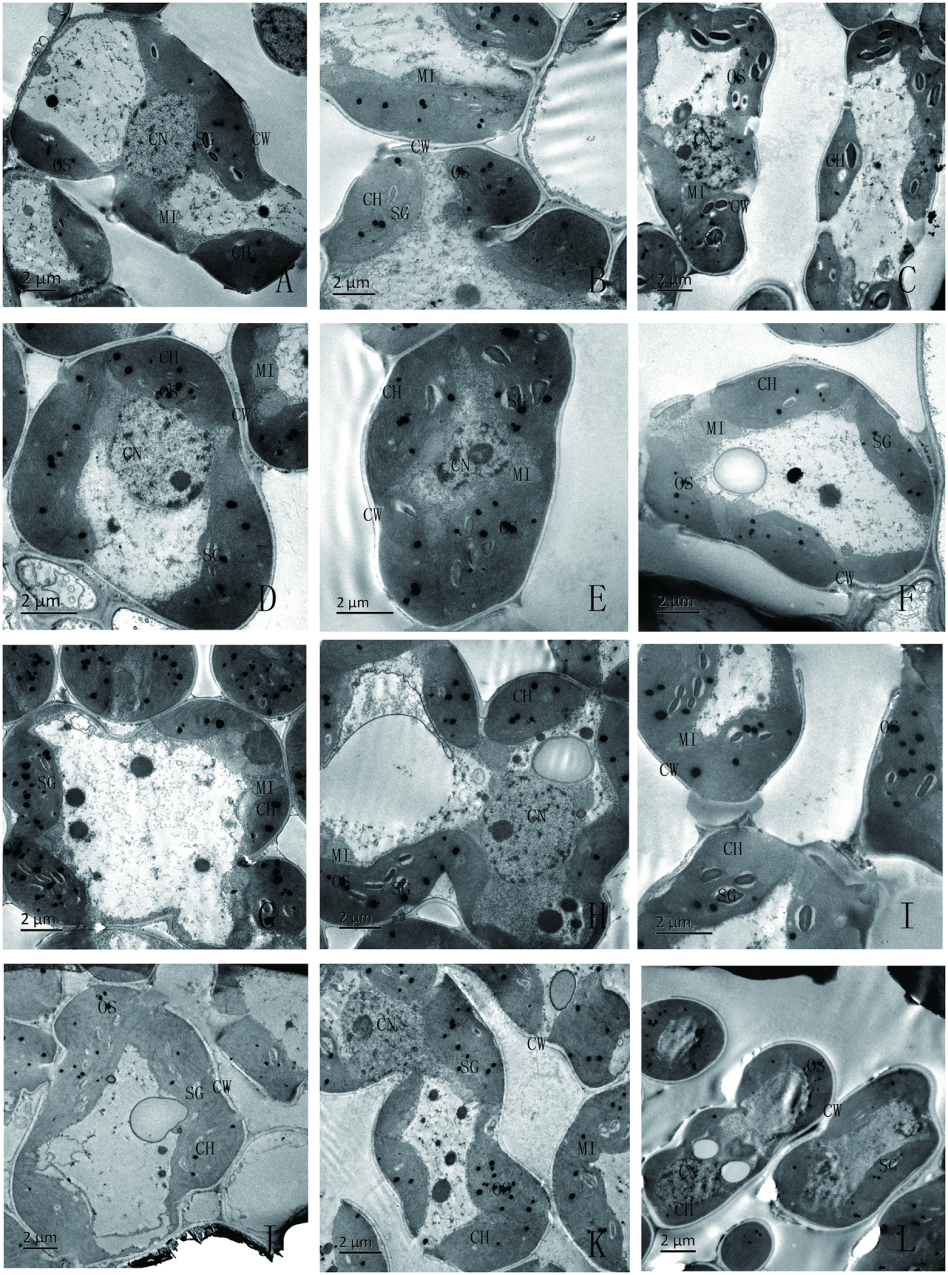
Mesophyll cell in rice seedling leaves under different treatments: (A-C) rain at pH 6.5; (D-F) SAR at pH 4.0; (G-I) SAR at pH 3.0; (J-L) SAR at pH 2.0; (A, D, G, J) no Si; (B, E, H, K) 2 mM Si; and (C, F, I, L) 4 mM Si. Symbols: CW: cell wall, SG: starch granule, OS: osmium, CH: chloroplast, CN: cell nucleus, MI: mitochondria.

The mesophyllcells of the rice seedlings treated with Si and SAR are shown in Fig 2A–2L. The mesophyll cells of the control were intact and included cell membranes, nuclei, chloroplasts and mitochondria (Fig 2A). The mesophyll cells of the rice seedlings exposed to the single 2 or 4 mM Si treatment were also intact (Fig 2B and 2C), but starch grains accumulated in large quantities in the chloroplasts of the rice seedling leaves treated with the single 4 mM Si solution. When the rice seedlings were treated with the SAR at pH 4.0, the mesophyll cell development was better, the cytoplasm was thicker, and the chloroplast and mitochondrial structures were more mature than in the control (Fig 2D). The mesophyll cells of the rice seedlings treated with 2 or 4 mM Si and SAR at pH 4.0 were intact. Specifically, the cytoplasm of the mesophyll cells that were treated with 2 mM Si and SAR was thicker, and the chloroplast and mitochondrial structures were more mature (Fig 2E and 2F). When the rice seedlings were treated with the single SAR at pH 3.0 (Fig 2G), the mesophyll cells were still intact, but the cytoplasm was thin, the chloroplasts were swollen, the granum thylakoid was thin, the lamellar structure of the thylakoid was loose, the number of starch granules and the amount of osmium were significantly greater, the matrix in the chloroplast was thin, and there were significantly more mitochondria than in the control. For the combined treatments with 2 or 4 mM Si and SAR at pH 3.0 (Fig 2H and 2I), the structures of the mesophyll cells, chloroplasts and mitochondria regained their shape to a certain extent; the number of mitochondria, starch granules and the amount of osmium were significantly decreased; the matrices in the chloroplasts and the cytoplasm in the mesophyll cells were thicker than those in the corresponding single SAR treatment. When the rice seedlings were treated with SAR at pH 2.0 (Fig 2J), some of the mesophyll cells and the chloroplast membranes were broken, the matrix flowed out, and the granum and lamellar structures of the thylakoid were deformed and collapsed. Moreover, the mesophyll cells of the rice seedlings treated with 2 mM Si and SAR at pH 2.0(Fig 2K)retained their integrity, and the chloroplasts were slightly swollen and had relatively clear granum and lamellar structures. The extent of the damage to the chloroplast and thylakoid was less than that of the treatments with the single SAR at pH 2.0. Themesophyll cells of the rice seedlings treated with 4 mM Si and SAR at pH 2.0 (Fig 2L) also retained their integrity, and the chloroplasts in the rice seedlings treated with 4 mM Si and SAR at pH 2.0 narrowed to an abnormal shape.

### Combined effects of Si and SAR on Si content of blade surfaces

**Fig 3:**
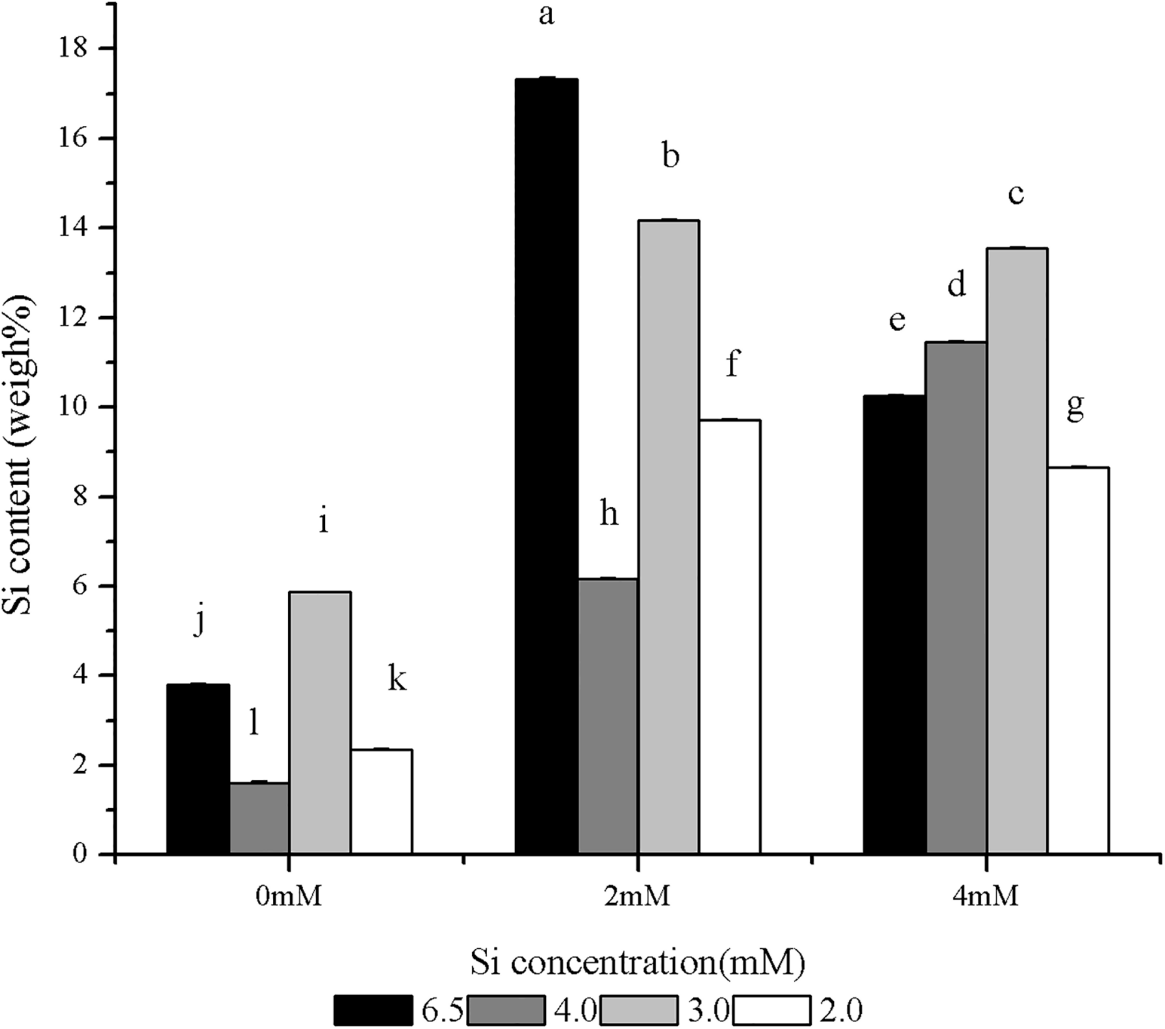
Effects of Si and SAR on Si content in rice leaves. n = 3, significantly differences at P < 0.05 were showed with different letter.

Fig 3 shows the effects of Si on the Si content in the epidermis under SAR stress. The content of Si in the leaves of rice seedlings treated with the single Si (2 or 4 mM) was significantly higher than that in the control. The content of Si in the leaves of the rice seedlings treated with the single 4 mM Si was decreased compared with that of the seedlings treated with the single 2 mM Si (Fig 3). Under the treatment with the single SAR at pH 4.0 or 2.0, the content of Si in the leaves was significantly decreased compared with the control. In contrast, the content of Si in the leaves of seedlings treated with the single SAR at pH 3.0 was significantly higher than that in the control (Fig 3).

The content of Si in the epidermis subjected to the combined treatments with 2 mM Si and SAR at pH 4.0, 3.0 or 2.0 were higher than those in the control leaves and the leaves subjected to the corresponding single SAR treatments, but the contents were decreased compared with those in the leaves treated with the single 2 mM Si treatments. The Si contents in the leaves of the rice seedlings treated with 4 mM Si and SAR at pH 4.0 or 3.0 were higher than those in the control leaves, the leaves subjected to the corresponding single SAR treatments and the leaves treated with the single 4 mM Si treatments. When the rice seedlings were treated with 4 mM Si and SAR at pH 2.0, the Si content in the leaves were higher than those in the control leaves, the leaves subjected to the corresponding single SAR treatments, but the contents were lower than those in the leaves treated with the single 4 mM Si solutions. The Si contents in the leaves of the rice seedlings treated with SAR at pH 3.0 were highest under the same concentration of Si. The two-way ANOVA results indicated an interaction between Si and SAR that affected the Si concentrations in the leaves of the rice seedlings treated with Si and SAR.

### Combined effects of Si and SAR on the WP densities of rice seedlings

**Fig 4:**
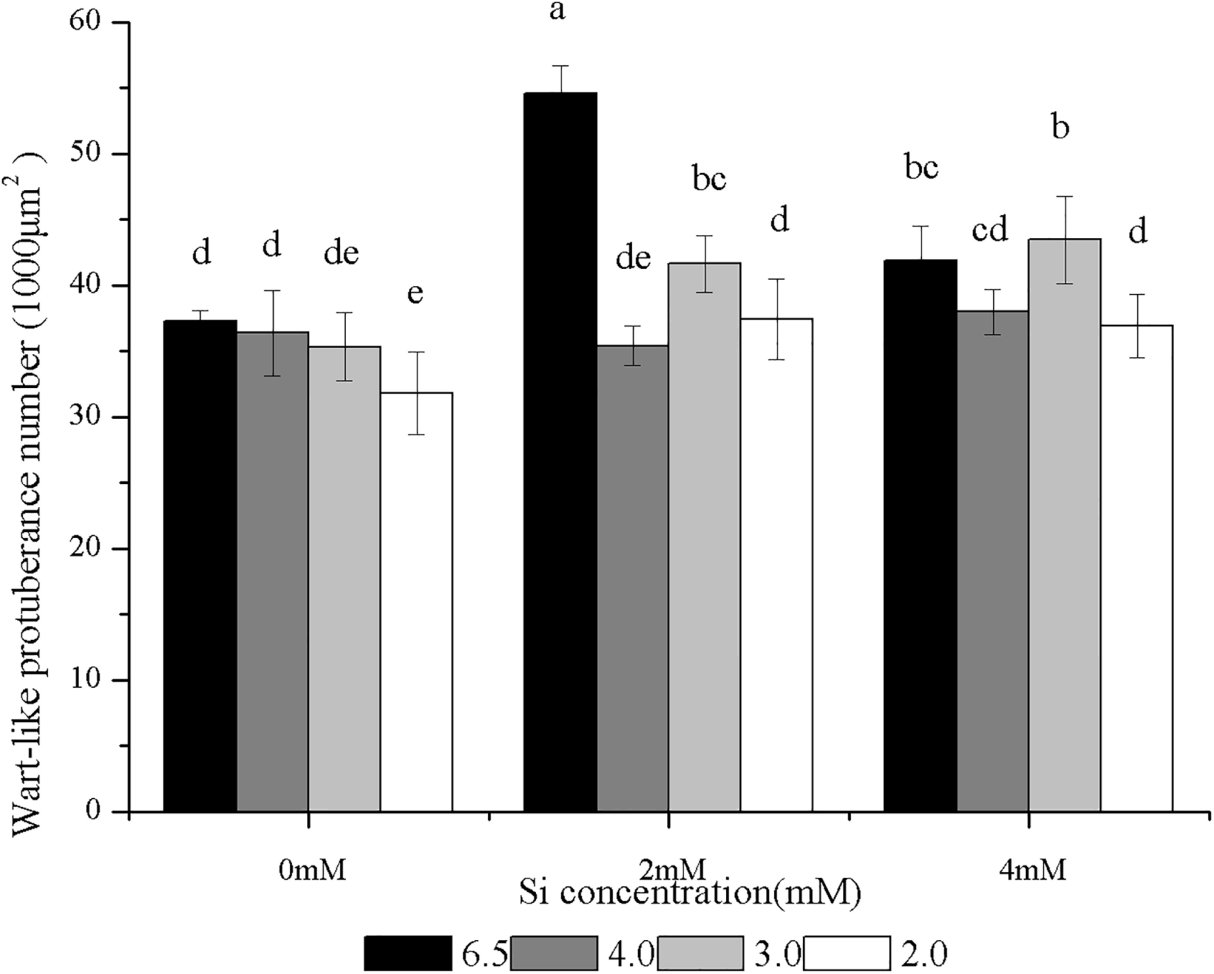
Effects of Si and SAR on wart-like protuberance (WP) number in rice leaves. n = 9, significantly differences at P < 0.05 were showed with different letter.

Fig 4 shows the effects of Si on the WP densities on the adaxial epidermis of the rice seedlings treated with SAR. The WP densities on the rice seedlings treated with the single Si (2 or 4 mM) were significantly higher than that in the control. The WP densities on the leaves of the rice seedlings treated with the single 4 mM Si were lower than that of the seedlings treated with the single 2 mM Si (Fig 4). The WP densities on the leaves treated with the single SAR at pH 4.0, 3.0 or 2.0 were lower than that in the control, but the reduction was not significantly different in the SAR at pH 4.0 or 3.0 treatments. The WP densities in the SAR at pH 2.0 treatment were significantly lower than that in the control (Fig 4).

The WP densities of the leaves subjected to the combined treatments with 2 or 4 mM Si and SAR at pH 4.0 were not significantly different from those of the control leaves or the leaves that were subjected to the single treatments with SAR at pH 4.0. However, the WP densities in the combined treatments were lower than that in the leaves treated with the corresponding Si-only solutions, and the WP densities in the 2 mM Si and SAR at pH 2.0 treatment were significantly lower than that of the single treatment with 2 mM Si The WP densities of the leaves of the rice seedlings treated with 2 or 4 mM Si and SAR at pH 3.0 were higher than those of the control leaves and the leaves subjected to the single treatments with SAR at pH 3.0. The WP densities in the 2 mM Si treatment at pH 3.0 were significantly lower than that of the single treatment with 2 mM Si. In the 4 mM Si treatment at pH 3.0, the WP densities were not significantly higher than that of the single treatment with 4 mM Si. When the rice seedlings were treated with 2 or 4 mM Si and SAR pH at 2.0, the WP densities were not significantly different from the control; however, they were significantly higher than that of the single treatment with SAR pH at 2.0 and significantly lower than that of the corresponding single treatment with Si. The WP density was positively correlated with the Si content of blade surfaces (Y = 0.9786X+30.6236, R = 0.65786, *p* = 8.33053E-4).

### Combined effects of Si and SAR on the epicuticular wax of rice seedlings

**Fig 5:**
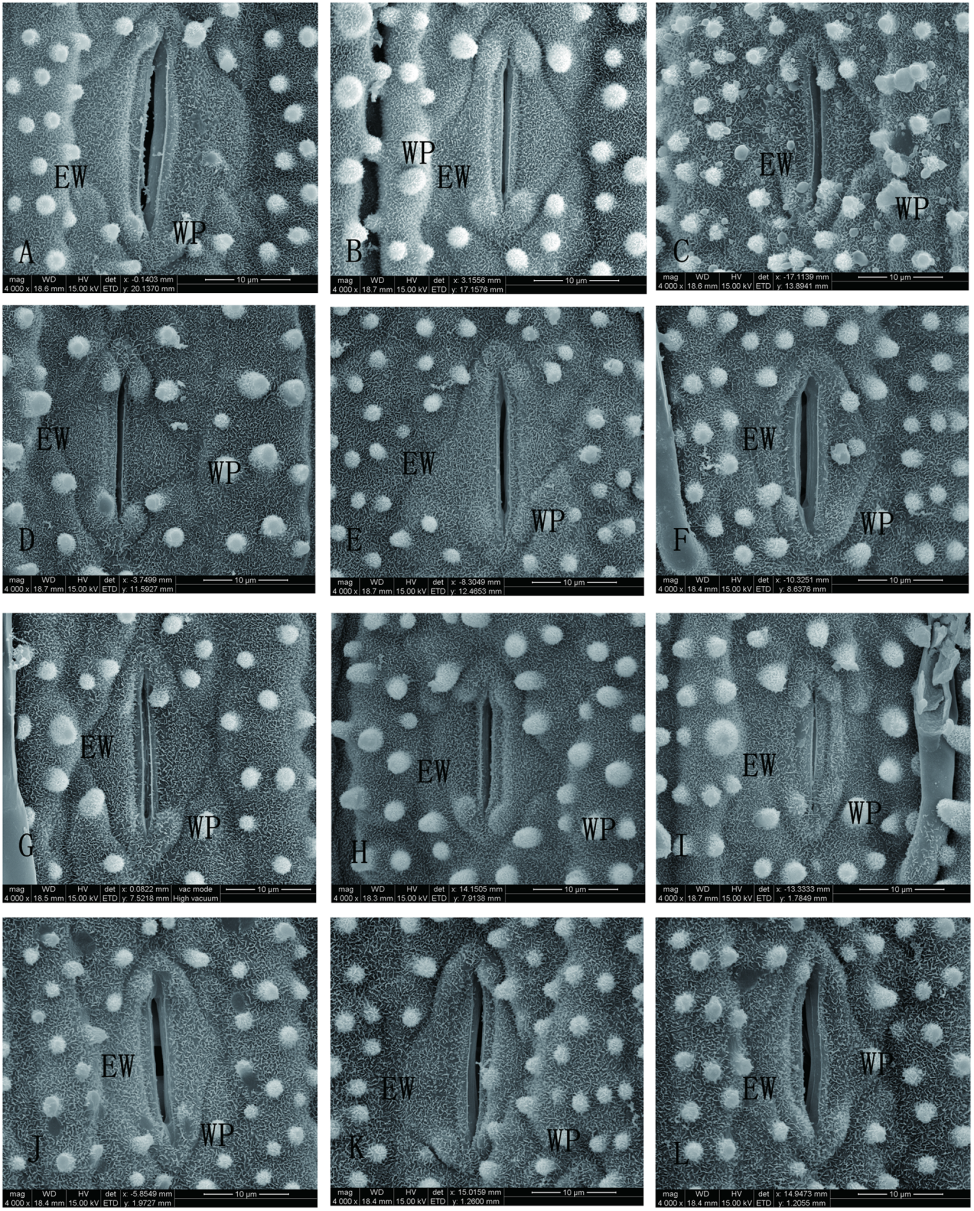
SEM of the adaxial epidermis structure in rice seedlings leaves under different treatments: (A-C) rain at pH 6.5; (D-F) SAR at pH 4.0; (G-I) SAR at pH 3.0; (J-L) SAR at pH 2.0; (A, D, G, J) no Si; (B, E, H, K) 2 mM Si; and (C, F, I, L) 4 mM Si. Symbols: WP: wart-like protuberance. EW: epicuticular wax.

The epicuticular wax on the middle parts of the second expanded adaxial blades of the rice seedlings treated with Si and SAR are shown in Fig 5A–5L. The observations indicated that the epicuticular wax of the control was rich and rod-like; however, it was rare on some WP (Fig 5A). The epicuticular wax was richer in the single treatments with 2 or 4 mM Si than that of the control (Fig 5B and 5C). The epicuticular wax of the rice seedlings under the single SAR treatment at pH 4.0 (Fig 5D) was decreased compared with that of the control. When the rice seedlings were treated with the SAR at pH 3.0 (Fig 5G), the epicuticular wax was increased compared with the control and the treatment with the single SAR at pH 4.0. When the pH values decreased to 2.0 (Fig 5J), the epicuticular wax was rarest in all treatments with SAR and the control.

When the rice seedlings were treated with Si (2 or 4 mM) and SAR at pH 3.0 or 2.0, the epicuticular wax was richer than that of the seedlings in the corresponding single SAR treatments. Under the same Si concentration conditions, the trends of the changes to the epicuticular wax resulting from increased pH values were similar to that of the single SAR treatments. Under the same pH values of SAR, the epicuticular wax of the rice seedlings treated with 2 Mm Si was richer than that of the rice seedlings treated with 4 Mm Si.

### Combined effects of Si and SAR on the blade stomata of rice seedlings

Table 1 shows the effects of Si onthe total stomatal density(tSD); the stomatal density (adSD), stomatal length (adSL), and stomatal width (adSW) of the adaxial epidermis; and the stomatal density (abSD), stomatal length (abSL), and stomatal width (abSW) of the abaxial epidermis of rice seedlings under SAR stress. The two-way ANOVA results indicated a significant interaction between Si and SAR that affected the six stoma parameters in the leaves of the rice seedlings treated with Si and SAR. The, abSL and abSW were obviously greater, the adSL and adSW were obviously lower, and the adSD, tSD and abSD were unchanged under the 2 mM Si treatments compared with those of the control. The tSD and abSD were obviously greater, the adSL and adSW were obviously lower, and the adSD, abSWand abSL were unchanged under 2 mM Si treatments compared with those of the control (Table 1).

**Table 1:** Effects of Si and SAR on stomatal features of leaf epidermis of rice seedling.

SAR (pH)	Si (mM)	Adaxial epidermis			Abaxial epidermis			Total stomatal density(mm^2^)
		Stomatal density(mm^2^)	Stomatal length (μm)	Stomatal width (μm)	Stomatal density(mm^2^)	Stomatal length(μm)	Stomatal width(μm)	
6.5	0	264.45+10.65 ef	28.23+1.80 abcde	16.10+0.54 ab	234.08+9.75 c	26.63+1.79 bcde	12.56+0.37d	498.53+19.71 def
	2	289.05+13.11 def	23.38+2.12 f	12.05+0.76 e	218.47+17.26 cde	30.11+1.91 a	14.93+0.98 bc	507.53+25.52 cdef
	4	269.03+13.38 ef	25.61+1.64 cdef	12.01+0.57 e	295.06+18.44 ab	24.94+1.92 de	13.28+0.66 cd	564.09+9.66 abcd
4.0	0	249.58+5.69 f	29.95+2.31 ab	16.06+0.76 ab	221.62+16.96 cde	28.16+1.32 abcd	16.80+0.82 a	471.20+14.78 f
	2	313.65+18.45 cd	28.70+1.95 abcd	15.67+0.61 abc	229.50+13.25 cd	26.96+1.55 abcde	14.96+0.76 abc	543.15+31.34 bcde
	4	338.25+9.34 abc	25.72+2.49 cdef	14.04+0.71 cd	261.66+19.72 abc	27.62+0.84 abcd	12.66+0.62 d	599.91+28.40 ab
3.0	0	360.87+12.44 ab	24.88+1.12 def	12.86+0.74 de	178.35+9.34 e	29.46+1.62 ab	14.65+0.98 bc	539.22+3.26 bcdef
	2	319.80+18.68 bcd	29.63+1.75 abc	16.38+0.96 ab	249.01+12.57 bc	26.90+0.68 abcde	15.83+1.16 ab	568.81+6.23 abc
	4	296.86+17.51 cde	24.42+1.44 ef	15.92+0.72 ab	184.50+16.90 de	24.22+1.56 e	12.64+0.67 d	481.36+30.36 ef
2.0	0	313.65+16.90 cd	25.90+2.11 bcdef	12.69+1.01 de	301.35+16.98 a	26.04+0.46 cde	12.74+1.02 d	615.01+33.45 a
	2	375.15+10.65 a	30.57+2.13 a	16.86+1.15 a	251.15+16.39 bc	28.57+2.03 abc	13.74+1.15 cd	626.30+26.99 a
	4	331.61+19.51 bcd	28.92+1.27 abcd	14.82+0.45 bc	288.99+21.51 ab	26.62+1.49 bcde	12.67+0.67 d	620.60+25.81 a
F		6.190	3.322	2.924	6.054	3.088	9.829	7.930
p		0.000*	0.011*	0.021*	0.000*	0.016*	0.000*	0.000*

Values are means ± standard deviation errors.

Significanty differences at *p*<0.05 were showed with different letter in the same line.

* Significance at 0.05 levels.

The tSD, adSD, adSL, adSW, abSD and abSL were unchanged, and the abSW was obviously greater under the SAR at pH 4.0 treatment than that in the control. The adSD, abSW were obviously greater, the adSW and abSD were obviously lower and the tSD, adSL and abSL were unchanged under the SAR at pH 3.0 treatment than those in the control. The tSD, adSD, and abSD were obviously greater, the adSW was obviously lower, and the abSL, adSL and abSW were unchanged under the SAR at pH 2.0 treatment compared with those of the control (Table 1).

For the combined treatments with 2 mM Si and SAR at pH 4.0, the adSD and tSD were significantly higher and the adSL, adSW, abSD, abSL and abSW had not significantly changed compared with the single treatment with SAR at pH 4.0. For the combined treatments with 4 mM Si and SAR at pH 4.0, the values of the six stoma parameters excluding abSW had not significantly changed, the abSW was obviously lower than those of the treatment with 2 mM Si and SAR at pH 4.0, while compared with the single treatment with SAR at pH 4.0, the tSD and adSD were obviously higher, the adSL, adSW and abSW were lower, and the abSD and abSL had not significantly changed. The values of the tSD, adSD and abSD gradually increased, the adSL, adSW and abSW had opposite trends, and the abSL gradually decreased but then increased with increasing Si concentrations under SAR at pH 4.0. For the combined treatments with 2 mM Si and SAR at pH 3.0, the adSL, adSW and abSD were markedly higher and the tSD, adSD, abSL and abSW had not obviously changed compared with the single treatment with SAR at pH 3.0. For the combined treatments with 4 mM Si and SAR at pH 3.0, the adSD, adSW and abSL had not obviously changed, the tSD, adSL, abSD and abSW were significantly lower than those of the treatment with 2 mM Si and SAR at pH 3.0. while compared with the single treatment with SAR at pH 3.0, the adSW was obviously higher, the adSD, abSL and abSW were significantly lower, and the tSD, adSL and abSD had not significantly changed. The values of the adSD and abSL gradually decreased, and adSL, adSW, abSD, abSW and tSD gradually increased but then decreased with increasing Si concentrations under SAR at pH 3.0. For the combined treatments with 2 mM Si and SAR at pH 2.0, the adSD, adSL and adSW were markedly higher, the abSD was significangly lower, and the tSD, abSL and abSW had not obviously changed compared with the single treatment with SAR at pH 2.0. For the combined treatments with 4 mM Si and SAR at pH 2.0, the adSD and adSW were obviously lower, while the abSL, tSD, adSL, abSD and abSW had not markedly changed compared with the 2 mM Si and SAR at pH 2.0. While compared with the single treatment with SAR at pH 2.0, the adSW was obviously higher, and the adSD, tSD, abSW, abSL, adSL and abSD and had not significantly changed. The abSD gradually decreased but then increased, while the values of the remaining six stoma parameters had opposite trends with increasing Si concentrations under SAR at pH 2.0. The two-way ANOVA results indicated an interaction between Si and SAR that affected the stomatal parameters in the leaves of the rice seedlings treated with Si and SAR.

The SEM observations showed that the stomata on the leaves of the rice seedlings treated with the single SAR at pH 4.0 were open, while the stomata on the rice seedlings treated with the single SAR at pH 3.0 or 2.0 were closed. When the pH of the SAR decreased to 2.0, the stomata were more closed and larger than those of the SAR at pH 4.0treatment. When Si was incorporated into the SAR at pH 4.0, 3.0 or 2.0, the stomata were open (Fig 5).

## Discussion

The phenotypes of the plants in this study showed obvious changes in response to acid rain, which damages the cuticles of the epidermis when indirect contact with plant surfaces and causes necrosis and chlorosis in leaves [44–48]. Similar histological and anatomical changes in the leaves were observed as a result of SAR treatments in our work (Fig 1). Applying exogenous Si can promote plant growth and relieve the deleterious effects of environmental stress [49,50]. Our experimental results showed similar conclusions (Fig 1). At the same time, effects of Si and SAR on the leaves of rice seedlings depended on the concentration of Si and the pH of the SAR (Fig 1). First, the leaves of the rice seedlings that were treated with a 2 mM concentration of Si were well developed, but the application of 4 mM Si obviously inhibits leaf growth (Fig 1). Second, spraying SAR at pH 4.0 promoted leaf growth and increased the areas of the leaves. When the rice seedlings were treated with SAR at pH 3.0, the rice leaves became smaller, thinner and showed small necrosis spots, and these symptoms were more significant when the leaves were sprayed with SAR at pH 2.0 (Fig 1). Third, the incorporation of a 2 mM concentration of Si into the SAR (pH 4.0) treatment increased the leaf area and thickness. The spraying of SAR at pH 4.0 had adverse effects in the 4 mM Si treatment. The incorporation of Si into the 3.0 or 2.0 pH SAR treatment decreased the number of necrosis spots and increased the leaf area, and the effect of the 2 mM Si treatment was better than that of the 4 mM Si treatment (Fig 1).

Mesophyll cells are the structural and functional units of plant leaves that contain many chloroplasts, mitochondria and various other organelles. Moreover, mesophyll cells are the most important part of photosynthesis, by which plants convert solar energy into chemical energy. In addition, photosynthesis is the basis of plant survival. Therefore, mesophyll cells have always been the focus of research, especially under environmental stress. The degree of damage to mesophyll cells is widely used to evaluate the intensity of stress. Our results from TEM indicated that the improvement/inhibition effects of Si and SAR on the leaves were closely related to the degree of development of the mesophyll cells (Figs 1 and 2). When rice seedlings were treated with the single 2 mM Si, the mesophyll cells were healthy (Fig 2B). However, the when the concentration of silicon was increased to 4 mM, the cytoplasm was thinner, and the volume and number of starch grains increased. One possible reason is that the application of 4 mM Si stressed the leaves of the rice seedlings to a certain extent and prohibited the photosynthetic products from being exported, which led to smaller leaves (Figs 1 and 2C). Healthier structures of the mesophyll cells were observed in the treatment with weak SAR (pH 4.0), which led to higher biomass and larger leaf areas (Figs 1 and 2D) than in the control. The incorporation of 2 mM Si into the 4.0 pH SAR treatment increased the chloroplast content, which led to higher biomass and larger and thicker leaves than inthe 4.0 pH SAR-only treatment (Figs 1 and 2E). The spraying of SAR at pH 4.0 on the rice seedlings treated with 4 mM Si improved the structure of the mesophyll cells, which led to larger leaves than in the 4 mM Si-only treatment (Figs 1 and 2F), which indicated that weak SAR (pH 4.0) could alleviate the stress from the 4 mM Si treatment. When the rice seedlings were treated with moderate SAR (pH 3.0), the cytoplasm was thinner, the structure of the chloroplasts was loose, the number of starch granules and the amount of osmium were significantly increased, the matrix in the chloroplast was thin, and the number of mitochondria were significantly increased and appeared in group form. Mitochondria are the “power plants” of cells and provide the energy for plant store pair the damage and overcome the effects of stress [51–55]. Large energy expenditures reduce biomass accumulation, which leads to smaller leaves (Figs 1 and 2G). More severe destruction of mesophyll cells was observed inthe rice seedlings treated with severe SAR (pH 2.0), which led to a greater reduction in the size of the leaves. The incorporation of Si (2 or 4 mM) with the moderate and severe SAR treatments improved the structure of the mesophyll cells compared with those observed following the corresponding treatments with SAR alone, and the improvements were accompanied by similar changes in leaf phenotypes.

Water absorption in plant leaves occurs viastomatal and non-stomatal pathways. A previous study showed that acid rain induced stomatal closure [48]. Therefore, the non-stomatal pathway may become the main channel for acid rain to enter the plant. Epicuticular wax is the first barrier to environmental stress in a plant [56,57]. The epidermal wax of the plant can limit the loss of non-stomatal water, decrease water permeability of the blade surface, and reduce moisture retention on the plant leaf surface [56–58]. Aprevious study showed that acid rain degraded epidermal wax [48,59], and our experiments showed similar results (Fig 5). Spraying SAR at pH 3.0promotes the formation of epidermal wax(Fig 5G); when the pH of the SAR decreased to 2.0, the epidermal wax of the rice leaf blades degraded (Fig 5J). It has been found that supplying Si can increase the epidermal wax content of leaves [21,60,61], and our experiments showed similar results (Fig 5B and 5C). However, the effect depended on the concentration of Si, and the 2 mM Si treatment was better than the 4 mM Si treatment. Our study showed that the addition of Si significantly increased the epidermal wax content under moderate or severe SAR (pH 3.0 or 2.0) stress compared with that under the corresponding single SAR treatment, and 2 mM Si treatment was better than the 4 mM Si treatment. This result indicated that Si treatment increases the epidermal wax on leaves, which may decrease the water permeability of rice leaves and reduce the moisture retention on the plant leaf surface, which eventually blocks the entry of acid rain solution. The induced increases in epidermal wax content on the leaf surfaces can be considered an important mechanism in the structure of the defence strategy against acid rain stress.

The hydrophobic property of the leaves is caused by the WPs and the wax of the leaves [62]. Our experiments showed that the changing trends of WP densities and Si on the surfaces of the leaves of the rice seedlings treated with Si (2 or 4 mM) and/or SAR (pH 4.0, 3.0 or 2.0) were similar to the trends of epidermal wax. At the same time, the correlation analysis performed in this study shows that the Si concentration on the surface of rice seedling leaves treated with Si and SAR is positively correlated with the WP density. That result indicated that Si treatment increases the WP density on leaves, which may also lead to a decrease in the water permeability of the rice leaves and reduce the moisture retention on the surface of the leaf, which will eventually block the entry of acid rain solution. The induced increases in the WP density on the leaf surfaces can be considered an important mechanism in the structure of the defence strategy against acid rain stress.

Stomata are the channels through which plants control water and gas exchange. Previous studies have shown that environmental stress leads to a change in stomatal density and size [63–67]. Supplying Si can change the stomatal density and size of a leaf to enhance the resistance of a plant [63,64, 68,69]. Previous studies have shown that higher stomatal densities and smaller stomatal sizes improved the sensitivity of the stomatal regulation of the plants and improved the stomatal function at similar leaf areas. Our two-way ANOVA results indicated an obvious interaction between Si and SAR that affected the stomatal characteristics, including stomatal density and size (Table 1). The 2 mM Si treatment increased the stomatal density and decreased the stomatal size of the adaxial epidermis, and the opposite trend was found in the abaxial epidermis. That result indicated that the stomatal function improved with the 2mM supply of Si. The 4 mM Si treatment decreased the stomatal size and increased the stomatal density of the abaxial epidermis, which indicated that the 4 mM supply of Si improved the stomatal function of the abaxial epidermis but restricted the stomatal function of the adaxial epidermis. The finding that supplying Si could induce a change in the stomatal characteristics was similar to a previous study by Yang [63]. SAR could also change the stomata characteristics. Compared with the control, the pH 4.0 SAR treatment increased the stomatal size of the abaxial epidermis and did not obviously change the stomata of the adaxial epidermis. The pH 3.0 SAR treatment increased the stomatal density and decreased the stomatal size of the adaxial epidermis, and the opposite trend was shown on the abaxial epidermis. The incorporation Si into SAR (pH 4.0, 3.0 or 2.0) led to changes in both the stomatal density and size. However, the induced changes showed an increase of total stomatal area (stomatal density×stomatal size). This increase in the area might be advantageous for gas exchange [70–73] but could also bed is advantageousas it would prevent acid rain from entering the plant body via the stomatal pathway. The results from Gao et al. [69] showed that changes in neither stomatal morphology nor stomatal density could explain the role of Si in decreasing the stomatal transpiration of maize plants. However, this study indicated that the incorporation of Si in the moderate and severe SAR (pH 3.0 or 2.0) treatments relieved the damage of acid rain on the stomata cells and maintained the structure and function of the stomata cells. Acid rain can also induce stomatal closure, but severe acid rain could damage and lead to the permanent opening of stomata cells. In our work, the SEM observations showed similar results. However, the stomata on the leaves of the rice seedlings treated with the SAR at pH 3.0 and Si were open. The above analysis indicated that under acid rain stress, the application of exogenous silicon could maintain the structure and function of stomata cells, and reduce the amount of acid rain entering the plant by the closing of stomata.

In summary, the effects of Si and acid rain on rice seedlings depended on the concentration of Si and the intensity of the acid rain. The light acid rain (pH 4.0) had stimulating effects, while the moderate and serious acid rain (pH 3.0 or 2.0) damaged the rice leaves. The incorporation of exogenous Si ameliorates the damage of the moderate and serious acid rain (pH 3.0 or 2.0) to the leaves by maintaining the structure and function of the mesophyll cells, increasing the epicuticular wax content and WP density, and improving the stomata characteristics in the leaves of rice seedlings.

## Supporting information

S1 File Si content. (SAV)

S2 File WP density. (SAV)

S3 File Stomatal parameters. (SAV)
